# Divergent anomaly in mesocorticolimbic dopaminergic circuits might be associated with different depressive behaviors, an animal study

**DOI:** 10.1002/brb3.808

**Published:** 2017-09-08

**Authors:** Mei Bai, Xiongzhao Zhu, Li Zhang, Yi Zhang, Liang Xue, Yuting Wang, Mingtian Zhong, Xiuwu Zhang

**Affiliations:** ^1^ Medical Psychological Institute Second Xiangya Hospital Central South University Changsha Hunan China; ^2^ Mental Health Institute of The Second Xiangya Hospital Key Laboratory of Psychiatry and Mental Health of Hunan Province Central South University Changsha Hunan China; ^3^ Department of Radiation Oncology School of Medicine University of Maryland Baltimore MD USA

**Keywords:** anhedonia, chronic unpredictable stress, DAT, depressive behaviors, dopamine receptor, explorary interest, maternal deprivation stress, mesocorticolimbic dopaminergic pathways, passive coping behavior, rodents

## Abstract

**Background:**

The mesocorticolimbic dopamine system, which originates from the ventral tegmental area (VTA) and projects primarily to the prefrontal cortex (PFC), olfactory tubercle (OT), nucleus accumbens (NAc), dorsal striatum (ST), and the amygdala (AMy), plays a pivotal role in determining individual motivation and sensitivity to rewards, namely, anhedonia. Not all depressive individuals exhibited anhedonia, thus, it is natural to speculate that the heterogenous manifestations of depression might be related to the mesocorticolimbic dopamine system. Maternal deprivation (MD) and chronic unpredictable stress (CUPS) are two well‐established depressogenic stressors, and they were proven to induce different depressive phenotypes.

**Methods:**

The depressive and anxiety‐like behaviors of MD and CUPS‐treated rats were measured by classical behavioral tests including open field, forced swimming, and sucrose preference test. The expression of D1‐5 dopamine receptors and DAT mRNA and protein in the mesocorticolimbic dopamine system of rats exposed to MD and CUPS were measured by real‐time PCR and Western blot, respectively.

**Results:**

Severe anhedonia was observed in MD but not CUPS rats. Divergent expression of D1 and D2 receptors and DAT mRNA and protein in the mesocorticolimbic dopamine system were found between MD and CUPS rats. Significant correlations between different depressive behaviors and D1‐/D2‐like receptors and DAT protein levels in the mesocorticolimbic dopamine system were observed.

**Conclusion:**

Different depressive behaviors of rats such as anhedonia, passive coping behavior, and declined exploratory interest might be related to divergent dopaminergic pathways. Anhedonia is associated with the dysfunction of VTA‐NAc and VTA‐OT dopaminergic pathways, the passive coping behavior is related to the dysregulation of VTA‐PFC and VTA‐AMy pathways, and individual exploratory interest is associated with abnormal activity of VTA‐PFC and VTA‐ST pathways.

## 1 INTRODUCTION

Depression is a highly heterogeneous mental illness that exhibits various clinical phenotypes (Bauer et al., 2007). Recent studies reported that merely 37–50% of individuals diagnosed with depression experience clinically significant anhedonia (Bogdan, Nikolova, & Pizzagalli, 2013; Pelizza & Ferrari, 2009). Our previous study in rats also demonstrated that the depressive phenotype induced by maternal deprivation (MD), a well‐verified depressogenic stressor, was characterized by severe anhedonia while the depressive phenotype triggered by chronic unpredictable stress (CUPS), another well‐established depressogenic stressor, primarily exhibited anxiety and passive coping behavior (Bai et al., 2012; Bai, Zhang, et al., 2014; Bai, Zhu, et al., 2014). However, the precise neurobiological basis of different depressive phenotypes remains unclear.

The mesocorticolimbic dopaminergic system, composed of dopaminergic neurons and their projections, has been proven to be involved in the reward‐motivated behaviors of individuals (Cohen, Haesler, Vong, Lowell, & Uchida, 2012; Ikemoto, 2007). The dopaminergic neurons are mainly located in the ventral tegmental area (VTA) (Beckstead, Domesick, & Nauta,^1979^) and project primarily to the prefrontal cortex (PFC), olfactory tubercle (OT), nucleus accumbens (NAc), dorsal striatum (ST), and the amygdala (AMy). These dopaminergic projections form five mesocorticolimbic dopaminergic neural pathways (Swanson, 1982).

Dopamine (DA) released from the terminal of the mesocorticolimbic dopaminergic system generally exerts effects via activating DA receptors. Five subtypes of dopamine receptors have been identified and classified into two groups: D1‐like receptors (including DRD1 and DRD5) and D2‐like receptors (including DRD2, DRD3, and DRD4) (Kebabian & Calne, 1979). D1‐like receptors are located mainly on postsynaptic membranes, and activation of D1‐like receptors results in an increase in intracellular cyclic adenosine monophosphate (cAMP) concentrations and subsequent activation of the associated neural pathways. D2‐like receptors reside on both presynaptic and postsynaptic membranes. Stimulation of D2‐like receptors reduces adenylate cyclase activity and subsequently inhibits related neural pathways (Stoof & Kebabian, 1984).

Recently, several lines of evidence consistently indicated that dysregulation of dopamine receptors and DAT in the mesocorticolimbic dopaminergic system were associated with depression (Eisch et al., 2003; Niwa et al., 2013; Winter et al., 2007). However, their roles in the emergence of different depressive phenotypes remain unclear. We hypothesized that MD and CUPS may induce divergent depressive phenotypes via exerting distinct effects on D1‐/D2‐like receptors and DAT in different mesocorticolimbic dopaminergic pathways. In this study, we measured the expression of D1‐/D2‐like receptors and DAT, in the VTA, PFC, OT, NAc, ST, and AMy of MD and CUPS‐treated rats and investigated their roles in the behaviors of rats exposed to different stressors.

## 2 MATERIALS AND METHODS

### 2.1 Animals

Ten Sprague–Dawley rats at the age of 3 months were provided by the Animal Center of Central South University and housed in polycarbonate cages under standard conditions in accordance with the Guide for Care and Use of Laboratory Animals (Chinese Council). After fertilization, rats were checked at 9:00 every day for delivery. Rat offspring born before 9:00 were designated as postnatal day 1 (PND 1). All experiments were conducted in accordance with an approved protocol from Central South University. Every effort was made to minimize the number and suffering of animals. Newborn male offspring from 10 pregnant rats were mixed and randomly divided into three groups: maternal deprivation group (MD, *N *= 8), chronic unpredictable stress group (CUPS, *N *= 8), and control group (C, *N *= 8). MD rats received maternal deprivation for 2 weeks after birth, CUPS rats received chronic unpredictable stress for 3 weeks after reaching 10 weeks old, and control rats received only standard husbandry care.

### 2.2 Maternal deprivation (MD)

The MD paradigm was carried out as previously described (Ploj, Romana, & Nylander, 2003). Briefly, rat offspring were separated from their mothers for 6 hours daily from PND 1 to PND 13 (the separations occurred at 9:00–15:00). To block communication between mothers and offspring, each offspring was placed in a new cell (32 cm × 32 cm × 14 cm, divided into four cells of the same size) covered with sawdust in a new room and later returned to their home cage when the experiment finished at 15:00 p.m. All experiments were conducted in a temperature‐controlled room (25°C). After PND21, rats were grouped and housed socially with the same gender until adulthood (10 weeks).

### 2.3 Chronic unpredictable stress (CUPS)

The CUPS paradigm was conducted following a previously established protocol (Willner, 1997) with small modifications. Rats at 10 weeks old were randomly exposed to one of the following stressors once daily for 3 weeks: food deprivation for 24 hr, water deprivation for 24 hr, home cages tilted horizontally at 30° for 24 hr (Pechlivanova, Tchekalarova, Nikolov, & Yakimova, 2010), electric foot shock for 20 s (800 mA, 1‐s duration, average 1 shock/10s), an elevated open platform (10 cm × 10 cm, 160 cm in height) for 2 hr (Storey et al., 2006), or restraint stress for 1 hr (Pechlivanova et al., 2010). Stress was given at different times of the day to establish unpredictability. The detailed procedures are shown in Table 1.

**Table 1:** The procedure of chronic unpredictable stress

	Week 1	Week 2	Week 3
Monday	Cage tilting	Electric foot shock	Elevated platform
Tuesday	Elevated platform	Cage tilting	Food deprivation
Wednesday	Electric foot shock	Restraint	Electric foot shock
Thursday	Food deprivation	Water deprivation	Cage tilting
Friday	Cage tilting	Elevated platform	Water deprivation
Saturday	Restraint	Food deprivation	Restraint
Sunday	Water deprivation	Restraint	Elevated platform

### 2.4 Sucrose consumption test

Sucrose consumption test was performed as described previously (Zhang et al., 2013, 2015). The whole test took 3 days. On day 1, rats were housed individually and given free access to two bottles of sucrose solution (1%, w/v and 100 ml). The cage was small and two identical bottles were used. Rats were trained to adapt to sucrose solution for 24 hr. On day 2, one bottle of sucrose solution was replaced with 100 ml of water for 24 hr. On day 3, rats were deprived of water and food for 23 hr, and then rats were given free access to two preweighed bottles of solution: 100 ml of sucrose solution (1%, w/v) and 100 ml of water. One hour later, the consumed liquid in both bottles was measured. The sucrose preference rate was calculated using the following formula: Sucrose preference rate = sucrose consumption / (water consumption + sucrose consumption) × 100%.

### 2.5 Open field test

Open field test was performed as previously described (Bai, Zhang, et al., 2014). The open field arena was made from an open rectangular plastic box (100 cm × 100 cm × 30 cm) with 25 squares (20 cm × 20 cm) painted on the floor. The 25 squares included 16 peripheral squares (along the wall of the box) and 9 central squares. At the time of the test, rats were coded by observers blind to the experimental design. Rats were then placed individually in the center of the field and allowed to explore the area freely for 5 min. The activity of the rats was recorded by an overhanging camera that was linked to a personal computer. Ethovision 3.0 (Noldus, The Netherlands) was applied to track the behaviors of the rats. The total distance a rat moved in the arena, distance a rat moved in the central squares, the number of vertical activity, and the number of fecal pellets present in the arena during the 5‐min test was recorded. The arena was cleaned with 70% alcohol between tests to make sure the current rat's behaviors were not affected by the imprint of previous rats. All behavioral tests were carried out on rats at the age of 13 weeks.

### 2.6 Forced swimming test

Forced swimming test was performed 24 hr after open field test following a previously established protocol (Weaver et al., 2005). Two swimming sessions were conducted: a 15‐min pretest on the first day followed by a 5‐min test the next day. At the time of the test, rats were placed individually in a Pyrex cylinder (21 cm × 46 cm) filled with 25°C water to a depth of 30 cm. After swimming for 15 min on day 1, rats were dried with towels and placed back in their home cage. The water in the cylinder was emptied and refilled between rats to ensure the rat's behaviors were not affected by a previous rat's scent. Twenty‐four hours after the first trial, rats were placed in the swimming apparatus again for a 5‐min test. A video camera hung above the cylinders was used to record the rats' activity. The immobility time (the time a rat spent in keeping its head above water with only slight movement) was recorded. The data were analyzed by two observers blind to the experimental conditions.

### 2.7 Sample collection

Animals were sacrificed after behavioral tests. Rats were anesthetized with intraperitoneal injections of pentobarbital sodium (50 mg/kg bodyweight), brains were rapidly removed from the skull, and VTA, PFC, OT, NAc, Amy, and ST tissues were immediately sectioned according to the anatomical atlas of Paxinos and Watson (Paxinos & Watson, 2005). Tissues were snap frozen in liquid nitrogen and kept at −80°C until use.

### 2.8 Real‐time reverse transcription quantitative PCR

Quantitative RT‐PCR was used to detect DRD1‐5 and DAT mRNA in VTA, PFC, OT, NAc, AMy, and ST tissues. Total RNA was extracted using Trizol reagent (Invitrogen, Carlsbad, CA, USA) by following the manufacturer's instructions. Reverse transcription was performed using RevertAid First‐Strand cDNA synthesis kit (MBI Fermentas, Burlington, ON, Canada) by following the manufacturer's protocol. cDNA was amplified using Roter‐Gene 3000 (Corbett Research, Sydney, NSW, Australia). The PCR primers are listed in Table 2. All qRT‐PCR reactions were run thrice.

**Table 2:** Real‐time PCR primer sequence

Target molecule	Forward	Reverse
DRD1	cttcgatgtgtttgtgtggttt	tcttccttcttcaggtcctcag
DRD2	cactcagatgcttgccattgttc	gtgggatgttgcaatcacagtgta
DRD3	tcaccctggatgtcatgatgtgta	gtgatcatgagtgccacacgtcta
DRD4	accccatcatctacaccatctt	agacatcagcggttctttcag
DRD5	gctacgagcgcaagatgac	attgagttggaccgggatgaag
DAT	gtactggcggctatgctggaa	gggtctgaaggtcacaatgctg
β‐actin	cacgatggaggggccggactcatc	taaagacctctatgccaacacagt

### 2.9 Tissue homogenizing and Western blot

The brain tissues were homogenized and Western blot was performed as previously described (Zhang et al., 2015). Ten microgram of total protein was applied to 10% SDS‐PAGE gel and transferred to polyvinylidene difluoride membranes (BioRad, Hercules, CA, USA). After blocking with blocking buffer for 1 hr, the membranes were incubated with primary antibody (DRD1, 1:150; DRD2, 1:100; DRD3, 1:1000; DRD4, 1:100; DRD5, 1:400; DAT, 1:300; Santa Cruz Bio. Inc., USA) overnight at 4°C. The membranes were then washed three times in Tris‐buffered saline containing 0.1% Tween 20 followed by incubation with horseradish peroxidase‐conjugated antimouse secondary antibody (1:4000; Santa Cruz Bio. Inc.) for 2 hr. The immune reactions were then visualized using an enhanced chemiluminescence kit (Millipore, USA). For loading control, the membranes were stripped and incubated with a GAPDH antibody (1:600). The densitometric analysis was performed using Bio‐Rad Quantity One software (Bio‐Rad, USA). All samples were run in duplicate on separate gels, and DRD1, DRD5, DRD2, DRD3, DRD4, DAT, and BDNF content was expressed relative to GAPDH.

### 2.10 Statistical analysis

Data were presented as mean and standard error of the mean and analyzed using the statistical package for the Social Sciences Version 17.0. The Levene test was used for assessment of variance homogeneity. One‐way analysis of variance (ANOVA) was used for the comparison among three groups if the variance of every group were homogeneous, and LSD test was used for the comparison between every two groups; Kruskal–Wallis H test was used for the comparison among three groups if the variance of every group were heterogeneous, and Nemenyi test was used for the comparison between every two groups. Correlations between gene expression and behavior indexes were analyzed using Pearson correlation. A *p *<* *.05 was considered statistically significant.

## 3 RESULTS

### 3.1 Effects of MD and CUPS on behavior of adult rats

Behavioral data obtained from open field test are presented in Table 3(A). Rats in the MD, CUPS, and control groups crawled similar total distances in the open field arena (*F*_2,21_ = 1.519, *p *= .242). Rats in the three groups showed significantly different number of vertical activity (*F*_2,21_ = 9.199, *p *= .001); the number of vertical activity was significantly decreased in MD and CUPS rats compared to control rats (*t *= −3.894, *p *=* *.001; *t *= −3.505, *p *=* *.002), but no difference was observed between MD and CUPS rats (*t *= −0.389, *p *=* *.701). There were significant differences in central area rate among the three groups (*F*_2,21_ = 10.587, *p *=* *.001); the central area rate in CUPS rats was significantly lower than that in MD and control rats (*t *= −4.599, *p *<* *.001); no difference in the central area rate was observed between MD and control rats (*t *= −2.043, *p *=* *.054). There was a significant difference in the amount of excrement left by rats in the three groups (*F*_2,21_ = 4.940, *p *=* *.017); CUPS rats left more excrement than MD and control rats (*t *= 2.508, *p *=* *.020; *t *= 2.893, *p *=* *.009), while MD and control rats left the same amount of excrement (*t *= 0.386, *p *=* *.703).

**Table 3:** Behavioral data in open field test, forced swimming test, and sucrose preference test from three groups ($\overset{¯}{x}$±SD)

	The total distance rats crawled (cm)	The number of vertical activity	The central area rate	The number of excrement
(A)				
MD group	532.16 ± 101.00	13.13 ± 3.44*	0.16 ± 0.04△	2.13 ± 1.13△
CUPS group	593.85 ± 104.64	13.88 ± 3.76*	0.11 ± 0.03*	3.75 ± 1.58*
C group	629.13 ± 130.01	20.63 ± 4.31	0.20 ± 0.05	1.88 ± 0.13
*F* value	1.519	9.199	10.587	4.940
*p* value	.242	.001	.001	.017

	Floating time (s)	Sucrose preference rate
(B)		
MD group	131.50 ± 38.02*, △	0.395 ± 0.139*, △
CUPS group	191.75 ± 40.69*	0.542 ± 0.131*
C group	92.88 ± 28.55	0.685 ± 0.129
*F* value	15.215	9.490
*p* value	.000	.001

(A)—behavioral data in open field test; (B)—behavioral data in forced swimming test and sucrose preference test; MD—maternal deprivation group; CUPS—chronic unpredictable stress group; C—control group; *—Compare to C group *p *< .05; △—compare to CUPS group *p* < .05.

The data from the forced swimming test and sucrose consumption test are presented in Table 3(B). Significant differences in immobility time were observed among three groups (*F*_2,21_ = 15.215, *p *< .001); MD and CUPS rats floated significantly longer than control rats (*t *= 2.138, *p *= .044;*t *= 5.473, *p *< .001), while CUPS rats floated significantly longer than MD rats (*t *= 3.335, *p *= .003). Significant differences in sucrose consumption were observed among three groups (*F*_2,21_ = 9.490, *p *= .001); both MD and CUPS rats consumed significantly less sucrose than control rats (*t *= −4.328, *p *< .001; *t *= −2.134, *p *= .043), while CUPS rats consumed significantly more sucrose than MD rats (*t *= 2.194, *p *= .039).

### 3.2 mRNA and protein expression in the VTA

**Figure 1:**
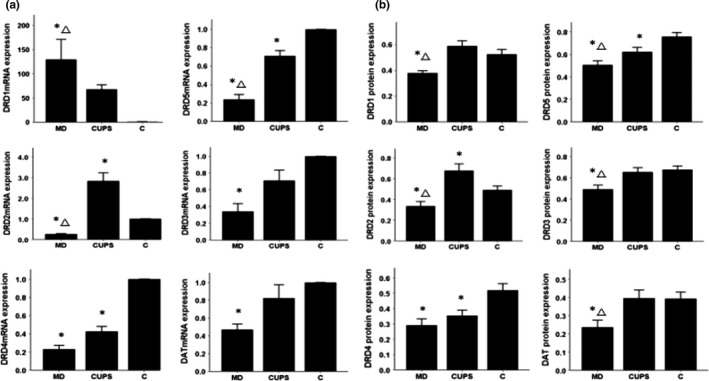
D1, D2 receptors, DAT mRNA, and protein level in VTA of rats from three groups. (a)—mRNA level , (b) —protein level; MD—maternal deprivation group, CUPS—chronic unpredictable stress group, C—control group; *—compare to C group *p* < .05; △ —compare to CUPS group *p* < .05; VTA—ventral tegmental area

As shown in Figure 1a,b, compared to control rats, the expression of DRD2‐5 and DAT mRNA and protein was significantly downregulated, DRD1 mRNA was upregulated, and DRD1 protein was downregulated in the VTA of MD rats. DRD2 mRNA and protein expressions were upregulated, and DRD5 mRNA and protein expressions were downregulated in the VTA of CUPS rats. DRD2 and DRD5 mRNA level and DRD1‐3 and DRD5 protein levels were significantly higher and DRD1 mRNA was significantly higher in the VTA of CUPS rats compared to MD rats.

### 3.3 mRNA and protein expression in the PFC

**Figure 2:**
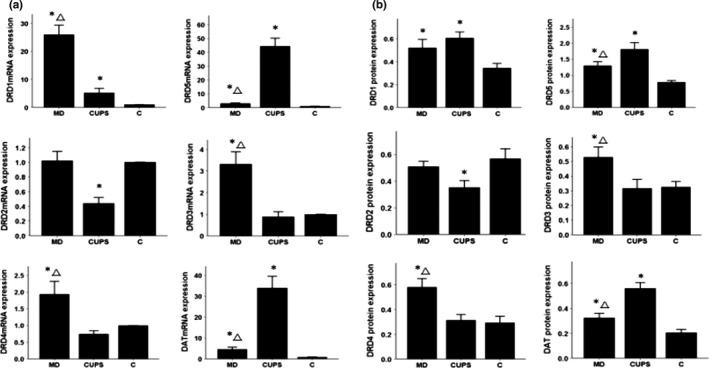
D1, D2 receptors, DAT mRNA, and protein level in PFC of rats from three groups. (a) —mRNA level ; (b) —protein level; MD—maternal deprivation group; CUPS—chronic unpredictable stress group; C—control group; *—compare to C group *p* < .05; △ —compare to CUPS group *p* < .05; PFC—prefrontal cortex

As shown in Figure 2(a,b), compared to control rats, DRD1, DRD3‐5, and DAT mRNA and protein levels were upregulated in the PFC of MD rats, DRD1, DRD5, and DAT mRNA and protein levels were upregulated and DRD2 mRNA and protein levels were downregulated in the PFC of CUPS rats. DRD3 and DRD4 mRNA and protein levels were higher, but DRD5 and DAT mRNA and protein levels were lower in the PFC of MD rats than in the PFC of CUPS rats.

### 3.4 mRNA and protein expression in the OT

**Figure 3:**
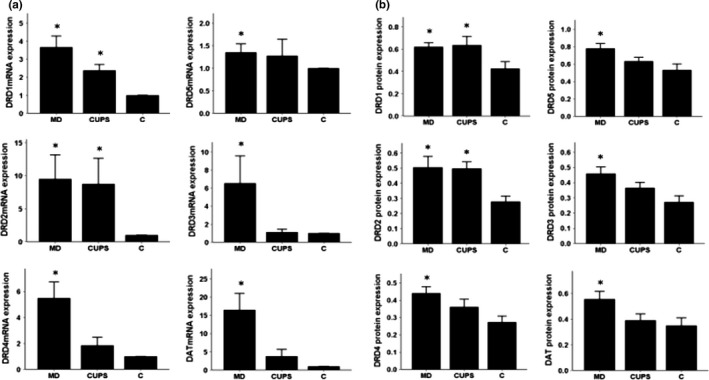
D1, D2 receptors, DAT mRNA, and protein level in OT of rats from three groups. (a) —mRNA level; (b) —protein level; MD—maternal deprivation group; CUPS—chronic unpredictable stress group; C—control group; *—compare to C group *p* < .05; OT—olfactory tubercle

As shown in Figure 3(a,b) , compared to control rats, DRD1‐5 and DAT mRNA and protein levels were upregulated in the OT of MD rats, DRD1‐2 mRNA and protein levels were upregulated in the OT of CUPS rats. No differences in DRD1‐5 and DAT mRNA and protein levels in the OT were detected between MD and CUPS rats.

### 3.5 mRNA and protein expression in the NAc

**Figure 4:**
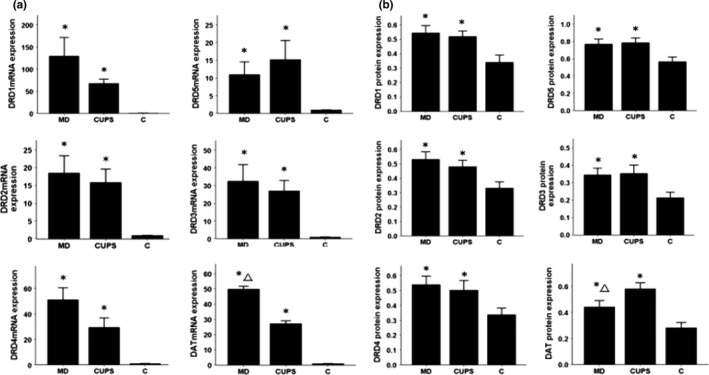
D1, D2 receptors, DAT mRNA, and protein level in NAc of rats from three groups. (a) —mRNA level; (b) —protein level; MD—maternal deprivation group; CUPS—chronic unpredictable stress group; C—control group; *—compare to C group *p* < .05; △—compare to CUPS group *p* < .05; NAc—nucleus accumben

As shown in Figure 4(a,b), compared to control rats, the DRD1‐5 and DAT mRNA and protein levels were increased in the NAc of MD and CUPS rats. DAT mRNA level was higher and protein level was lower in the NAc of MD rats than in the NAc of CUPS rats.

### 3.6 mRNA and protein expression in the AMy

**Figure 5:**
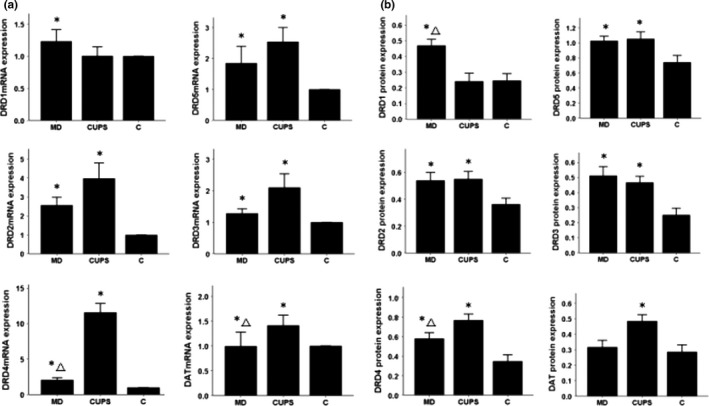
D1, D2 receptors, DAT mRNA, and protein level in AMy of rats from three groups. (a) —mRNA level; (b) —protein level; MD—maternal deprivation group; CUPS—chronic unpredictable stress group; C—control group; *—compare to C group *p* < .05; △—compare to CUPS group *p* < .05; AMy—amygdala

As shown in Figure 5(a,b), compared to control rats, the DRD1‐5 mRNA and protein levels were increased in the AMy of MD rats. The DRD2‐5 and DAT mRNA and protein levels were upregulated in the AMy of CUPS rats. DRD4 and DAT mRNA and DAT protein levels were higher in the AMy of CUPS rats than in the AMy of MD rats. DRD1 protein levels were lower in the AMy of CUPS rats than in the AMy of MD rats.

### 3.7 mRNA and protein expression in the ST

**Figure 6:**
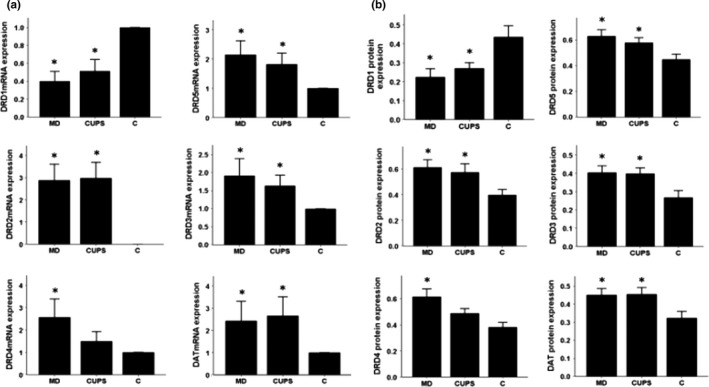
D1, D2 receptors, DAT mRNA, and protein level in ST of rats from three groups. (a) —mRNA level; (b) —protein level; MD—maternal deprivation group; CUPS—chronic unpredictable stress group; C—control group; *—compare to C group *p* < .05; ST—striatum

As shown in Figure 6(a,b), compared to control rats, the DRD2‐5 and DAT mRNA and protein levels were upregulated and DRD1 protein was downregulated in the ST of MD rats. The DRD2‐3, DRD5, and DAT mRNA and protein levels were increased and the expression of DRD1 mRNA and protein was decreased in the ST of CUPS rats. No difference in DRD1‐5 and DAT mRNA and protein levels in the ST was detected between MD and CUPS rats.

### 3.8 The correlation analysis between DRD1‐5 and DAT protein levels and the behavioral indexes of rats

As shown in Table 4(A), the number of vertical activity of rats positively correlated with the DRD3‐5 protein levels in the VTA and DRD1 protein level in the striatum. The number of vertical activity of rats negatively correlated with DRD2‐4, and DAT protein levels in the striatum and DRD5, DAT protein level in the PFC.

**Table 4:** The correlation analysis between behavioral data and DRD1‐5, DAT protein levels in VTA, ST, PFC, AMy, NAc, and OT of rats

	DRD1	DRD5	DRD2	DRD3	DRD4	DAT
(A)						
VTA	0.143	0.548**	0.259	0.487*	0.429*	0.049
ST	0.878**	0.201	−0.882**	−0.790**	−0.672**	−0.685**
PFC	−0.377	−0.481*	0.199	−0.214	−0.264	−0.493*
(B)						
AMy	−0.054	0.152	0.466*	0.482*	0.413*	0.453*
PFC	0.466*	0.668**	−0.347	−0.117	−0.077	0.703**
(C)						
VTA	0.252	0.606**	0.316	0.479*	0.337	0.563**
NAc	−0.345	−0.281	−0.517**	−0.712**	−0.695**	−0.679**
OT	−0.506*	−0.423*	−0.458*	−0.599**	−0.439*	−0.677**

(A)—The correlation analysis between the number of vertical activity and DRD1‐5, DAT protein levels in VTA, ST, PFC of rats; (B)—The correlation analysis between the immobility time and DRD1‐5, DAT protein levels in AMy, PFC of rats; (C)—The correlation analysis between the sucrose preference rate and DRD1‐5, DAT protein levels in VTA, NAc, OT of rats; VTA—ventral tegmental area; ST—striatum; PFC—prefrontal cortex; AMy—amygdala; NAc—nucleus accumben; OT—olfactory tubercle; *—*p* < .05; **—*p* < .01.

As shown in Table 4(B), the immobility time positively correlated with DRD2‐4 and DAT protein levels in the amygdala and DRD1, DRD5, DAT protein levels in the PFC.

As shown in Table 4(C), the sucrose preference rate of rats positively correlated with DRD3, DRD5, and DAT protein levels in the VTA, and negatively correlated with DRD2‐4, and DAT protein levels in the NAc, and DRD1‐5 and DAT protein levels in the OT.

## 4 DISCUSSION

Anhedonia is one of the cardinal elements for the diagnosis of a depressive episode (American Psychiatric Association. 2007). However, recent reports revealed that only 37%–50% of individuals diagnosed with depression experience clinically significant anhedonia (Bogdan et al. 2013; Pelizza & Ferrari, 2009). This implicates that depression could be subdivided into at least two subtypes, with or without anhedonia. This viewpoint is also supported by animal studies showing that MD but not CUPS induce severe anhedonia in rats (Bai, Zhang, et al., 2014). MD and CUPS also exert dissimilar effects on the expression of D1, D2‐like receptors, and DAT in some of the mesocorticolimbic dopaminergic pathways (Bassareo, De Luca, & Di Chiara, 2007; Brenes & Fornaguera, 2008). In this study, we first demonstrated that different depressive behaviors of rats such as anhedonia, passive coping behavior, and exploratory interest may be related to divergent dopaminergic pathways. Anhedonia was found to be associated with the dysfunction of VTA‐NAc and VTA‐OT dopaminergic pathways, the passive coping behavior was found to be related to the dysregulation of VTA‐PFC and VTA‐AMy dopaminergic pathways, and individual exploratory interest has correlation with the activity of VTA‐PFC and VTA‐ST dopaminergic pathways.

The results from the behavioral tests gave suggestions as our previous findings (Bai, Zhang, et al., 2014), that is, CUPS, an adulthood stress, was prone to trigger anxiety and more severe passive coping behavior in rats than MD, while MD, an early life stress, led to more severe anhedonia (viz., seemed to be more harmful to rats' sensitivity to reward) than CUPS. Given anhedonia was previously reported as a valid predictor of poor response to antidepressant treatment (McMakin et al.,^2012^) and adversity during childhood was more frequently observed in patients with treatment‐resistant depression than patients with treatment‐sensitive depression (Tunnard et al., 2014). It could be naturally speculated that the depression induced by early life stress tends to exhibit treatment resistance, and this needs to be assessed in further studies.

Results from recent studies indicated that different types of chronic stress might exert different effects on the sufferers' central nervous system (Valenti, Gill, & Grace,^2012^; Venzala, Tordera, García‐ García, & Elizalde,^2013^). For example, Valenti found that chronic mild stress inhibited, while chronic social stress increased, the activity of dopaminergic neurons in the VTA (Valenti et al.,^2012^). Venzala's study demonstrated that chronic mild stress and social stress exerted inconsistent effects on various neurotransmitters' levels in many brain regions (Venzala et al.,^2013^). In this study, MD and CUPS induce dissimilar alterations in rats' mesocorticolimbic dopaminergic system. For instance, compared to control rats, MD‐treated rats exhibited higher DRD1‐5 and DAT expression while CUPS‐treated rats exhibited only higher DRD1‐2 expression in the OT. Additionally, compared to CUPS‐treated rats, MD‐treated rats exhibited lower DRD1‐3 and DRD5 gene expression in the VTA, higher DRD3, DRD4 and lower DRD5, DAT protein level in the PFC, higher DAT protein level in the NAc, and higher DRD1, lower DRD4, DAT expression in the AMy. These results strongly suggested that different depressogenic stressors might exert divergent effects on mesocorticolimbic dopaminergic circuitry.

**Figure 7:**
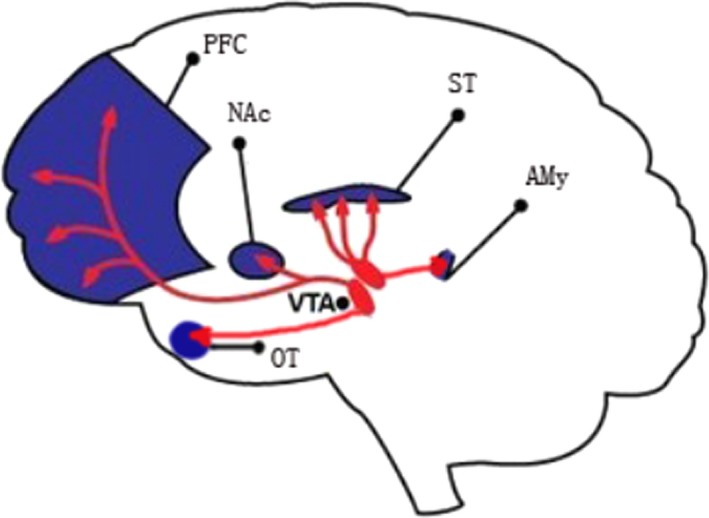
Five mesocorticolimbic dopaminergic pathways focused in this study. VTA—ventral tegmental area; PFC—prefrontal cortex; OT—olfactory tubercle; NAc—nucleus accumbens; AMy—amygdala; ST—striatum

Previous studies demonstrated that the dopamine activity in the dorsal striatum, nucleus accumbens, prefrontal cortex, and amygdala might determine individual response to stimulus, sensitivity to reward, working memory, and emotional learning, respectively (Dalley, Everitt, & RSTbins, 2011; Phillips, Salussolia, & Hitchcott, 2010; Saunders & RSTinson, 2012). This study demonstrated that different depressive behaviors in the stress‐treated rats were associated with the activity of different mesocorticolimbic dopaminergic neural pathways (as shown in Figure 7).

Firstly, the vertical activity of rats in the open field test, reflecting rats' exploratory interest level, showed linear associations with only DRD3‐5 levels in the VTA; DRD1‐4 and DAT levels in the ST; and DRD5 and DAT levels in the PFC, which suggested that the VTA‐PFC and especially the activity of VTA‐ST dopaminergic pathways might primarily determine individual exploratory interest level. Both the stress‐treated MD and CUPS rats showed less exploratory interest, elevated DAT and DRD2‐4 levels, and reduced DRD1 level in the ST. As DRD2‐4 are inhibitory receptors and DRD1 is excitatory receptor (Jackson & Westlind‐Danielsson, 1994), the upregulation of DRD2‐4, DAT and the downregulation of DRD1 in the ST could synergically result in a decline in the activity of VTA‐ST neural pathway, which might finally lead to individual decreased exploratory interest. In contrast, the upregulation of excitatory DRD5 receptor in the ST of both MD‐ and CUPS‐treated rats might not be the direct effect of MD or CUPS, but might be the compensatory response toward the decreased activity of the VTA‐ST neural pathway.

Secondly, this study revealed that passive coping behavior, which was reflected by the immobility time of rats in the forced swimming test, was associated with DRD2‐4, DAT levels in the AMy, and DRD1, DRD5, and DAT levels in the PFC. These results suggest that individual passive coping behavior might be mainly determined by the activity of VTA‐PFC and especially VTA‐AMy dopaminergic pathways. Both MD and CUPS rats showed more severe passive coping behavior and elevated DRD2‐4 levels in the AMy than control rats. On the other hand, the CUPS‐treated rats behaved more passively than MD‐treated rats. This might be due to the less active VTA‐AMy pathway in CUPS rats than in MD rats, reflected by the higher DRD4 and DAT levels in the AMy of CUPS rats compared to MD rats. In contrast, the upregulation of DRD1 and DRD5 excitatory receptors in the AMy of both the stress‐treated rats might be a compensatory response toward the reduced activity of VTA‐AMy neural pathway.

It was suggested in previous studies (Simona & Stefano, 2012) that increased VTA‐NAc DA activity might trigger the emergence of active coping strategies when an event appraised as a stressor and that decreased VTA‐NAc DA activity is the underlying basis of passive coping with unescapable/uncontrollable stressful situations; for example, Pascucci et al. found that the novel and unescapable/uncontrollable stressor initially induced an increase in tonic levels of DA in NAc and then led to a decrease in DA level in NAc even below prestress levels which lasted as long as the stressful situation (Nicniocaill & Gratton, 2007; Pascucci, Ventura, Latagliata, Cabib, & Puglisi‐Allegra,^2007^; Ventura, Cabib, & Puglisi‐Allegra, 2002). This different response corresponds with the process of the primary and secondary appraisal of a stressor which cannot be removed, escaped, or predicted by the organism, and NAc DA fluctuations are modulated by the medial prefrontal cortex, which is also involved in stress appraisal (Pascucci et al.,^2007^). The results from this study suggesting the reduced VTA‐AMy and VTA‐PFC DA activity involved in passive coping behavior might be an interesting supplement to fully understand the biological foundation of passive and active coping strategies.

Finally, anhedonia indicated by the sucrose preference rate correlated with DRD3, DRD5, DAT levels in the VTA, DRD2‐4, and DAT levels in the NAc, and DRD1‐5 and DAT levels in the OT. This result suggested that anhedonia might be predominantly determined by the activity of VTA‐NAc and VTA‐OT dopaminergic pathways. Both MD and CUPS rats exhibited more severe anhedonia; elevated DRD2‐4, and DAT levels in the NAc; and elevated DRD2 level in the OT compared to control rats. The upregulation of DRD2‐4, DAT in the NAc, and DRD2 in the OT could collaboratively reduce neuronal activity of VTA‐NAc and VTA‐OT pathways, which might subsequently reduce sucrose consumption in stressed rats. In addition, MD‐treated rats exhibited more severe anhedonia than CUPS‐treated rats. This might result from the less active VTA‐NAc and VTA‐OT pathways in MD rats than in CUPS rats due to the higher DAT level in the NAc of MD rats compared to CUPS rats and elevated DRD3, DRD4, and DAT levels in the OT of MD rats. Moreover, the upregulation of DRD1 and DRD5 in the NAc and OT of both MD‐ and CPUS‐treated rats might be a compensatory response toward the decline in neuronal activity of VTA‐NAc and VTA‐OT pathways.

An intriguing study from Susan Andersen et al. (Nadja, Britta, Kai, Shirisha, & Susan, 2016) has proven that upregulation or blocking DRD1 overexpression in the PFC could increase or decrease sucrose preferences rate in rats, which demonstrated that elevated DRD1 expression in the PFC increased hedonic behavior, whereas blocking DRD1 overexpression in the PFC resulted in anhedonic behavior. This interesting finding together with the findings in our study suggest that anhedonia might be resulted from the declined activity of two dopaminergic VTA‐NAc and VTA‐OT pathways. This might be modulated by the activity of DRD1 in the PFC, but it needs to be determined in further studies.

The results of this study suggested that chronic stress during both early life and adulthood might exert distinct effects on rat behaviors as well as the expression of DA receptors and DAT in different DA neural pathways and different depressive behaviors might be determined by different DA neural circuitries. However, there are several limitations to this study. Firstly, the mechanisms by which chronic stresses regulate the expression of these genes have not been profoundly explored in this study. Another limitation is that we only explored the dopaminergic mechanisms underlying anhedonia, passive coping behavior, and exploratory interest but have not explored many other types of depressive behaviors, such as social withdrawal, irritability, psychomotor retardation, and so on (Dournes, Beeské, Belzung, & Griebel,^2013^).

In conclusion, this study suggests that different psychological stressors, such as MD and CUPS, induced divergent depressive performances in rats through dissimilar effects on neural activity of the dopaminergic pathways. In addition, the depressive behaviors of anhedonia, passive coping behavior, and declined interest might be associated with the activity of VTA‐NAc, VTA‐OT, VTA‐AMy, and VTA‐ST dopaminergic neural pathways, respectively.

## CONFLICT OF INTEREST

All authors declared no conflict of interest.
