# Supplementary material for: Divergent anomaly in mesocorticolimbic dopaminergic circuits might be associated with different depressive behaviors, an animal study
Source: Brain Behav. 2017 Sep 8;7(10):e00808. doi: 10.1002/brb3.808 (PMC5651392; doi:10.1002/brb3.808)
Supplement: Supplementary file 1 [file BRB3-7-e00808-s001.docx]

**1.The detailed statistical information for text of 3.2 was listed below:**

As shown in Fig. 1, compared to control rats, the expression of DRD2-5 and DAT mRNA ($X^{2}$=16.000，*p*<0.001; $X^{2}$=16.000，p<0.001; $X^{2}$=16.000，*p*<0.001; $X^{2}$=16.000，p<0.001; $X^{2}$=16.000，*p*<0.001)and protein (t=-2.153，*p*=0.042; t=-3.382，*p*=0.003; t=-4.000，*p*=0.001; t=-4.667，*p*<0.001)was significantly downregulated, DRD1 mRNA （$X^{2}$=16.000，*p*<0.001）was upregulated, and DRD1 protein （t=-3.042，*p*=0.006）was downregulated in the VTA of MD rats. DRD2 mRNA（$X^{2}$=16.000，*p*<0.001） and protein（t=2.569，*p*=0.017）expressions were upregulated, and DRD5 mRNA ($X^{2}$=16.000，*p*<0.001)and protein(t=-2.50，*p*=0.021 )expressions were downregulated in the VTA of CUPS rats. DRD2 and DRD5 mRNA level （$X^{2}$=16.000，*p*<0.001; $X^{2}$=12.444，*p*<0.001）and DRD1-3 and DRD5 protein（t=4.375，*p*<0.001; t=4.736，*p*<0.001; t=2.945，*p*=0.008; t=2.148，*p*=0.045） levels were significantly higher and DRD1 mRNA was significantly higher in the VTA of CUPS rats compared to MD rats.

**2.The detailed statistical information for text of 3.3 was listed below:**

As shown in Fig.2, compared to control rats, DRD1, DRD3-5, and DAT mRNA($X^{2}$=16.000，*p*<0.001; $X^{2}$=16.000，*p*<0.001; $X^{2}$=12.444，*p*=0.001;$X^{2}$=16.000，*p*<0.001;$X^{2}$=16.000，*p*<0.001) and protein（t=2.415，*p*=0.025；t=2.506，*p*=0.021；t=3.556，*p*=0.002；t=2.490，*p*=0.021；t=2.245，*p*=0.036）levels were upregulated in the PFC of MD rats, DRD1, DRD5, and DAT mRNA （$X^{2}$=16.000，*p*<0.001；$X^{2}$=16.000，*p*<0.001；$X^{2}$=16.000，*p*<0.001）and protein（t=2.291，*p*=0.032；t=4.976，*p*<0.001；t=6.698，*p*<0.001）levels were upregulated and DRD2 mRNA（$X^{2}$=16.000，*P*<0.001） and protein（t=-2.667，*p*=0.014）levels were downregulated in the PFC of CUPS rats. DRD3 and DRD4 mRNA（$X^{2}$=9.000，*p*=0.008；$X^{2}$=9.000，*p*=0.008） and protein（t=2.617，*p*=0.016；t=3.296，*p*=0.003） levels were higher, but DRD5 and DAT mRNA（$X^{2}$=16.000，*p*<0.001；$X^{2}$=16.000，*p*<0.001） and protein（t= -2.485，*p*=0.021；t= -4.452，*p*=0.000）levels were lower in the PFC of MD rats than in the PFC of CUPS rats.

**3.The detailed statistical information for text of 3.4 was listed below:**

As shown in Fig.3，compared to control rats，DRD1-5 and DAT mRNA （$X^{2}$=16.000，*P*<0.001；$X^{2}$=12.444，*p*=0.001；$X^{2}$=7.273，*p*=0.021；$X^{2}$=16.000，*P*<0.001；$X^{2}$=16.000，*P*<0.001）and protein（t=2.241，*p*=0.037；t=2.974，*p*=0.007；t=3.224，*p*=0.004；t=3.000，*p*=0.007；t=2.988，*p*=0.007；t=2.524，*p*=0.019） levels were upregulated in the OT of MD rats, DRD1-2 mRNA（$X^{2}$=16.000，*p*<0.001；$X^{2}$=9.600，*p*=0.006） and protein （t=2.414，*p*=0.025；t=2.547，*p*=0.009）levels were upregulated in the OT of CUPS rats. No differences in DRD1-5 and DAT mRNA（$X^{2}$=1.000，*p*=0.952；$X^{2}$=1.000，*p*=0.952；$X^{2}$=1.000，*p*=0.952；$X^{2}$=1.000，*p*=0.952；$X^{2}$=0.000，*p*=1.000；$X^{2}$=4.000，*p*=0.137） and protein（t=-0.172，*p*=0.862；t=0.092，*p*=0.925；t=1.621，*p*=0.119；t=1.411，*p*=0.174；t=1.771，*p*=0.092；t=2.024，*p*=0.055）levels in the OT were detected between MD and CUPS rats.

**4.The detailed statistical information for text of 3.5 was listed below:**

As shown in Fig.4, compared to control rats，the DRD1-5 and DAT mRNA （MD:$X^{2}$=16.000，*p*<0.001；$X^{2}$=9.600，*p*=0.006；$X^{2}$=16.000，*p*<0.001；$X^{2}$=16.000，*p*<0.001；$X^{2}$=16.000，*p*<0.001；$X^{2}$=16.000，*p*<0.001；CUPS:$X^{2}$=16.000，*p*<0.001 for all） and protein（MD：t=3.091，*p*=0.005；t=3.062，*p*=0.006；t=2.330，*p*=0.030；t=2.590，*p*=0.018；t=2.671，*p*=0.014；t=2.540，*p*=0.020；CUPS: t=2.712，*p*=0.012;t=2.292，*p*=0.033;t=2.485，*p*=0.022;t=2.115，*p*=0.047; t=2.882，*p*=0.009; t=4.746，*p*<0.001） levels were increased in the NAc of MD and CUPS rats . DAT mRNA（ $X^{2}$=16.000，*p*<0.001）level was higher and protein（t= -2.206，*p*=0.040）level was lower in the NAc of MD rats than in the NAc of CUPS rats.

1. **The detailed statistical information for text of 3.6 was listed below:**

As shown in Fig.5, compared to control rats，the DRD1-5 mRNA（$X^{2}$=9.600，p=0.006；$X^{2}$=16.000，p<0.001；$X^{2}$=7.273，p=0.021；$X^{2}$=12.444，p=0.001；$X^{2}$=9.600，p=0.006）and protein（t=3.431，p=0.002；t=2.299，p=0.031；t=3.714，p=0.001；t=2.578，p=0.017；t=2.415，p=0.025）levels were increased in the AMy of MD rats. The DRD2-5 and DAT mRNA （$X^{2}$=16.000，p<0.001；$X^{2}$=9.600，p=0.006；$X^{2}$=16.000，p<0.001；$X^{2}$=12.444，p=0.001；$X^{2}$=12.444，p=0.001）and protein（t=2.442，p=0.023；t=3.071，p=0.006；t=4.667，p<0.001；t=2.636，p=0.016；t=3.210，p=0.004）levels were upregulated in the AMy of CUPS rats . DRD4 and DAT mRNA（$X^{2}$=16.000，p<0.001；$X^{2}$=9.000，p=0.008） and DAT protein（t=2.710，p=0.013）levels were higher in the AMy of CUPS rats than in the AMy of MD rats. DRD1 protein（t=-3.508，p=0.002）levels were lower in the AMy of CUPS rats than in the AMy of MD rats .

**6.The detailed statistical information for text of 3.7was listed below:**

As shown in Fig.6, compared to control rats，the DRD2-5 and DAT mRNA（$X^{2}$=12.444，*p*=0.001；$X^{2}$=7.273，*p*=0.021；$X^{2}$=9.600，*p*=0.006；$X^{2}$=12.444，*p*=0.001；$X^{2}$=9.600，*p*=0.006） and protein （t=2.654，*p*=0.015；t=2.740，*p*=0.013；t=3.640，*p*=0.002；t=2.935，*p*=0.008；t=2.462，*p*=0.023）levels were upregulated and DRD1 protein （t=-3.239，*p*=0.004）was downregulated in the ST of MD rats. The DRD2-3, DRD5 and DAT mRNA （$X^{2}$=16.000，*p*<0.001；$X^{2}$=9.600，*p*=0.006；$X^{2}$=12.444，*p*=0.001；$X^{2}$=9.600，*p*=0.006）and protein（t=2.173，*p*=0.042；t=2.620，*p*=0.017；t=2.113，*p*=0.047；t=2.538，*p*=0.019）levels were increased and the expression of DRD1 mRNA（$X^{2}$=16.000，*p*<0.001）and protein （t=-2.537，*p*=0.019）was decreased in the ST of CUPS rats . No difference in DRD1-5 and DAT mRNA（$X^{2}$=1.000，*p*=0.952；$X^{2}$=0.000，*p*=1.000；$X^{2}$=1.000，*p*=0.952；$X^{2}$=0.000，*p*=1.000；$X^{2}$=0.000，*p*=1.000；$X^{2}$=0.000，*p*=1.000） and protein（t=-0.702，*p*=0.490；t=0.481，*p*=0.636；t=0.120，*p*=0.907；t=1.984，*p*=0.062；t=0.823，*p*=0.419；t=-0.077，*p*=0.938）levels in the ST was detected between MD and CUPS rats.

**7.The detailed statistical information for text of 3.8 was listed below:**

As shown in Table 4(A), the number of vertical activity of rats positively correlated with the DRD3-5 protein levels in the VTA（r=0.420，*p*=0.041；r=0.487，*p*=0.016；r=0.548，*p*=0.006）and DRD1 protein level in the striatum (r=0.878, *p*<0.001). The number of vertical activity of rats negatively correlated with DRD2-4, and DAT protein levels in the striatum（r=-0.882，*p*<0.001；r=-0.790，*p*<0.001；r=-0.672，*p*<0.001；r=-0.685，*p*<0.001）and DRD5, DAT protein level in the PFC（r=-0.481，*p*=0.017；r=-0.493，*p*=0.014）.

As shown in Table 4(B), the immobility time positively correlated with DRD2-4 and DAT protein levels in the amygdala（r=0.466，*p*=0.022；r=0.482，*p*=0.017；r=0.413，*p*=0.045；r=0.453，*p*=0.026）and DRD1, DRD5, DAT protein levels in the PFC （r=0.466，*p*=0.022；r=0.688，*p*<0.001；r=0.703，*p*<0.001).

As shown in Table 4(C), the sucrose preference rate of rats positively correlated with DRD3, DRD5, and DAT protein levels in the VTA （r=0.479，*p*=0.018；r=0.606，*p*=0.002；r=0.563，*p*=0.004）, and negatively correlated with DRD2-4, and DAT protein levels in the NAc（r=-0.517，*p*=0.010；r=-0.712，*p*<0.001；r=-0.695，*p*<0.001；r=-0.679，*p*<0.001）, and DRD1-5 and DAT protein levels in the OT（r=-0.506，*p*=0.012；r=-0.458，*p*=0.024；r=-0.599，*p*=0.002；r=-0.439，*p*=0.032；r=-0.423，*p*=0.039；r=-0.677，*p*<0.001）.
